# El mural de Fernando Oreste Nannetti en el Hospital Psiquiátrico de Volterra, Italia: análisis iconográfico

**DOI:** 10.1590/S0104-59702025000100015

**Published:** 2025-05-02

**Authors:** Pedro Trujillo Arrogante

**Affiliations:** i Investigador postdoctoral, Instituto de Historia/Centro de Ciencias Humanas y Sociales/Consejo Superior de Investigaciones Científicas. Madrid – España. pejotrujillo@gmail.com

**Keywords:** Psiquiatría, Italia, Arte, Mural, Fernando Oreste Nannetti (1927-1994, Psychiatry, Italy, Art, Mural, Fernando Oreste Nannetti (1927-1994

## Abstract

El artículo analiza el mural realizado por Fernando Oreste Nannetti en el Hospital Psiquiátrico de Volterra (Italia), durante su ingreso entre 1958 y 1973. Para ello, el texto se estructura en siete apartados, incluyendo la introducción y las consideraciones finales, que estructuran las líneas principales: el segundo y el tercero se destinan a presentar al autor y el Hospital Psiquiátrico de Volterra; entre el cuarto y el sexto, se elabora un conciso análisis iconográfico del mural. El objetivo es conectar estos contenidos con la biografía y los intereses o inquietudes de Nannetti a partir de los dos temas principales: su experiencia en el sistema médico psiquiátrico y su interés por la ciencia y la tecnología.

## Abstract (en)

This article analyzes the mural made by Fernando Oreste Nannetti at the Volterra Psychiatric Hospital (Italy), during his stay between 1958 and 1973. The text introduces the author and the Volterra Psychiatric Hospital and then develops a concise iconographic analysis of the mural. It aims to connect the contents with Nannetti’s biography, and the interests or concerns based on the two main themes: his experience in the psychiatric medical system and his interest in science and technology.

El espacio psiquiátrico ha sido testigo de manifestaciones artísticas poco comunes en los libros de historia del arte: aquellas realizadas por un grupo reducido de pacientes, que comenzaron a ser estudiadas en la segunda mitad del siglo XIX desde una óptica científico-médica (Trujillo Arrogante, 2023). En un principio, los objetivos que motivaron estos estudios fueron ampliar los peritajes psiquiátricos y los diagnósticos de las enfermedades mentales, entre cuyos autores cabe citar a Auguste Tardieu (1818-1879), Paul Max-Simon (1837-1889), Cesare Lombroso (1835-1909) o Andrea Cristiani (1862-1948). En el siglo XX surgieron otras técnicas en el espacio clínico psiquiátrico que ligaban al paciente con la creación plástica, como son la laborterapia o la arteterapia. También es el periodo en que, y a raíz de la influencia vanguardista, este tipo de manifestaciones conquistaron de manera paulatina la valoración artístico-estética; los primeros trabajos publicados en esta línea surgen a principios de siglo y aún pertenecían a autores del ámbito psiquiátrico, como Paul Meunier (1873-1957), Hans Prinzhorn (1886-1933) o Walter Morgenthaler (1882-1965); entre 1920 y 1940 ya hubo una serie de figuras del tejido artístico y cultural europeo interesadas por su potencial creativo, cuyo colofón se dio en 1945 con la creación del *art brut* ^1^ por el artista francés Jean Dubuffet (1901-1985).

En el presente trabajo abordamos un objeto de estudio en el marco de este tema: la obra mural realizada por Fernando Oreste Nannetti (1927-1994) en el Hospital Psiquiátrico de Volterra (Italia) durante su ingreso entre 1958 y 1973. Los trabajos precedentes dedicados al mural se agrupan en tres labores principales: documentación, ubicada entre 1978 y principios de este siglo y destinada a conservar el mural debido a su fragilidad material – incisiones sobre una pared compuesta de cal y arena; inspiración e interpretación, llevada a cabo por otros creadores – tanto plásticos como audiovisuales –, que a su vez dieron vida a obras derivadas de un diálogo con el mural; e investigación, sobre todo en la línea del *art brut* y los discursos artísticos, de lo que cabe citar a Lucienne Peiry, directora de la Collection d’Art Brut de Lausana y autora de una exposición y varias publicaciones sobre el mural (Peiry, 2011, 2020).

Partiendo de estos trabajos y el estudio de las imágenes que se conservan del mural, en los siguientes apartados nos centraremos en la conexión entre el autor y la obra, algo que, en ocasiones, se ha tratado de un modo somero. Esta labor la llevamos a cabo desde un enfoque que combina la historia de la psiquiatría con la historia del arte, con el objetivo de mostrar que las influencias estéticas y los principales contenidos del mural están ligadas a las vivencias y las inquietudes del autor. En especial, con sus experiencias dentro del sistema sanitario italiano y el interés por la ciencia y la tecnología, ligado esto último a su desarrollo profesional previo a la hospitalización.

## Fernando Oreste Nannetti: esbozo biográfico

Fernando Oreste Nannetti nació en Roma, en 1927. La poca información que los investigadores han reunido sobre su biografía se ciñe al contexto médico, es decir, las fuentes primarias proceden de los ingresos en distintas instituciones. Las causas que le vinculan a este ambiente se identifican ya desde los primeros años de vida, pues su infancia y adolescencia estuvieron marcadas por diversos traumas: el padre abandonó a la familia – se desconoce su identidad –, y de la madre, Concetta Nannetti – de quien adopta el apellido –, apenas existe información. El abandono paterno puso a Concetta en una difícil situación económica, obligándola a internar a Fernando entre 1934 y 1936, primero en una organización de la caridad y luego en un centro psiquiátrico para menores. En este periodo, y partiendo de los autores que han consultado las fuentes clínicas (Trafeli, Trafeli, Manoni, 1985; Trafeli, Peiry, Miorandi, 2017; Peiry, 2020), no se revela ningún diagnóstico, lo que nos lleva a considerar el ingreso psiquiátrico como una acción todavía relacionada con los problemas económicos.

Hasta 1942 existe un vacío documental. En ese año, Nannetti aparece de nuevo en la escena institucional sanitaria tras ingresar en el Ospedale di Carlo Forlanini di Roma debido a una enfermedad reumática de la columna vertebral. Los fuertes dolores y la completa incapacitación derivados de la enfermedad hicieron de esta una de sus experiencias más traumáticas, la cual se vio agravada por el segundo abandono, ahora de la madre. Concetta nunca regresó a por Fernando, razón por la que, solo y menor de edad, hubo de quedarse en el hospital hasta 1944 (Batini, 2007).

Entre 1944 y 1948 tiene lugar otro vacío biográfico, pero sabemos que durante estos años se ganó la vida como electricista, una profesión vinculada con algunos de los fragmentos del mural en Volterra. En 1948 fue procesado por desacato a la autoridad, lo que condujo a su primer peritaje psiquiátrico, cuyo diagnóstico concluyó con un “vizio totale di mente” – trastorno mental – y, por consiguiente, se le recluyó diez años en el pabellón judicial del Ospedale Psichiatrico di Santa Maria della Pietà di Roma. Durante este periodo, los informes clínicos ya revelan una esquizofrenia. Además, en este periodo el comportamiento de Nannetti era disruptivo, rebelde y ruidoso, distinto de la personalidad introvertida y tranquila que se mostró años después en el Ospedale Psichiatrico di Volterra, al cual fue trasladado en 1958 (Peiry, 2020, p.17-19).

En Volterra ingresó de nuevo en la sección judicial, el pabellón Ferri, donde estuvo hasta 1961; después pasó unos años en la sección Charcot y en 1968 volvió al Ferri. En cuanto a las relaciones personales durante este internamiento, fuera de la institución no conserva familiares o amistades, pero escribe cartas a personajes inventados (Batini, 2007). Dentro, solo se relacionó con Aldo Trafeli, un celador a quien consideró su único amigo. De hecho, y como veremos más adelante, los testimonios de Trafeli son la fuente principal para estudiar a Nannetti, pues ha permitido revelar los secretos que guarda el mural (Trafeli, Trafeli, Manoni, 1985; Trafeli, Peiry, Miorandi, 2017; Trafeli, Quirici, 2022).

Antes de publicarse la ley 180/1978, comúnmente conocida como Ley Basaglia (Pasquale, 2011), en 1973 Nannetti fue trasladado a su última institución psiquiátrica, el Istituto Bianchi de Volterra, donde permaneció hasta morir, en 1994.

## Volterra: de la Sección de Demencia al Hospital Psiquiátrico

Volterra es una pequeña ciudad italiana de origen etrusco que se sitúa entre Pisa y Siena, en la región de Toscana. La identidad histórica ha pervivido a lo largo del tiempo gracias a su patrimonio arqueológico, el cual ha convertido a la ciudad en uno de los principales centros de investigación sobre la civilización etrusca (Sorge, 2019).

A las afueras del entramado urbanístico, en lo alto de una colina al sudeste se levanta un imponente complejo arquitectónico de finales del siglo XIX y principios del XX. Es el Ex Ospedale Psichiatrico, cuyo origen se encuentra en la Sección de Demencia del Ricovero di Mendicità – asilo de pobres –, abierta en 1888 por la Congregación de la Caridad aprovechando las dependencias del antiguo convento de San Girolamo. Nueve años más tarde, la expansión de esta sección llevó a separarla administrativamente del Ricovero, obteniendo así su autonomía legal y la denominación de Asilo Dementi – Asilo para Enfermos Mentale. A principios del siglo XX, la institución experimentó otro cambio administrativo de acuerdo con el reale decreto de 5 de junio de 1902, y a petición de Michelangelo Inghirami, presidente en aquel entonces de la Congregación, por el que adquirió la categoría de manicomio con el nombre de Frenocomio di S. Girolamo. El primer director del frenocomio fue Luigi Scabia (1868-1934), una figura destacada en la historia de la psiquiatría clínica italiana, quien venía de ejercer como médico en los manicomios de S. Clemente de Venecia y Quarto dei Mille de Génova. Durante su dirección en Volterra, Scabia acometió varios cambios, entre los que cabe mencionar la última reforma administrativa en 1933, que otorgó al Frenocomio la categoría de Ospedale Psichiatrico dependiente de la Prefettura di Pisa; y, por otro lado, abrir la institución a enfermos de cualquier procedencia – no solo de la región – y en cualquier estado, incluidos peligrosos y crónicos (Fiorino, 2011, p.41-75). Para esta medida, se proyectó la mayor ampliación de la institución en todos sus años de existencia, con la cual se logró erigir una verdadera ciudad de treinta mil metros cuadrados edificados sobre cinco millones de superficie total (Trafeli, Quirici, 2022, p.31-39).

Las instalaciones se dividían entre las zonas residenciales y las clínicas, y, conforme a la idea de crear una institución autónoma y autosuficiente, también se incluyeron servicios, como un matadero, una panadería, una zapatería, una carpintería, una herrería y una sastrería, entre otros. La mayoría de aquellos puestos de trabajo fueron desempeñados por los propios pacientes como parte de un sistema de laborterapia, una actividad terapéutica que empezó a ser frecuente en las instituciones psiquiátricas a mediados del siglo XX (Sanín, 1976; Vanadia, 2021).

Scabia también trató de establecer alternativas asistenciales y modelos culturales con el objetivo de superar el aislamiento y el encierro manicomial, de lo que cabe destacar otras iniciativas, junto a la laborterapia, como el *open door* – puertas abiertas – y la llamada colonización psiquiátrica o de alienados. Algo para lo que, y según el modelo que crea en Volterra, probablemente tome como referencia la comunidad de Gheel en Bélgica (Huertas, 1988): los pacientes de Volterra salían del hospital, respiraban aire libre, caminaban por las calles y saludaban a los vecinos. Incluso, participaban en la economía de la ciudad y de sus entornos, pues fueron puestos a cargo de familias con negocios a las que ayudaban a cambio de formar parte de un hogar, aprender un oficio y recibir un sueldo – un porcentaje de este iba para el hospital a modo de pago por la pensión completa. Lejos de manifestar algún tipo de recelo hacia este programa, según Trafeli y Quirici (2022, p.42) los volterranos se mostraron comprensivos, incluso “orgullosos” de participar en una comunidad tan dinámica, innovadora y positiva para el paciente.

Al igual que otros psiquiatras europeos – y de la misma época – interesados por las terapias con implicaciones culturales, sirva de ejemplo el francés Auguste Marie (Couet, 2017), Scabia incorporó actividades como música, pintura, danza, lectura o jardinería (Manicomio…, s.f.). Tras su muerte, en 1934, hubo varios intentos por continuar con estas actividades, e, incluso, implementarlas. Entre todas, la más significativa fue organizada por el economista e historiador volterrano Enrico Fiumi (1908-1976), quien en la década de 1950 contrató a pacientes del hospital para participar en las excavaciones arqueológicas de la ciudad. Estas campañas tuvieron un enorme peso en la historiografía etrusca y, por consiguiente, en convertir a Volterra en uno de los principales centros de investigación de esta civilización. Tal es así, que en la actualidad se conserva una placa conmemorativa otorgada por la ciudad a los participantes, entre los que se menciona al hospital psiquiátrico (Manicomio…, s.f.; Trafeli, Quirici, 2022, p.42-48).

En cuanto a la división de las instalaciones, la estructura de Volterra estuvo organizada en *padiglioni* – pabellones –, entre los que se distribuían los pacientes según el tipo de enfermedad o condición legal de ingreso. Una de las secciones más significativas fue el Padiglione Ferri, compuesto por un edificio de dos plantas y un enorme patio, y donde Nannetti estuvo ingresado la mayoría de sus años. Ferri, en tanto que era el pabellón judicial, estaba cercado por un muro rematado con alambre de espino y vigilado día y noche mediante torres de control. El pabellón comenzó a construirse en 1930 y se destinó a pacientes altamente “peligrosos”, con una capacidad inicial de mil residentes. Por motivos de seguridad, se aisló de otras secciones y del exterior – no gozaban de las libertades antes descritas. Según el testimonio de Aldo Trafeli, el ambiente del pabellón solía ser tranquilo, aunque hubo ocasiones en que hubo de intervenir la policía y el personal enviado por la Prefettura di Pisa (I graffiti…, 2002). En el patio acontecía la vida diaria, donde los presos-pacientes se entretenían jugando a la petanca o a las cartas, durmiendo, fumando, leyendo o hablando. Mientras tanto, Nannetti trabajaba con ahínco en el mural (Peiry, 2020, p.9-14).

Tras la muerte de Scabia, Giovanni De Nigris asumió la dirección en 1934 e inauguró una política sanitaria de técnicas y metodologías que en esos años comenzaban a ser frecuentes por los psiquiátricos de Italia, como la malarioterapia, la insulinoterapia, el electroshock y la lobotomía, las cuales eran, sin lugar a duda, menos amables para el paciente que las iniciativas de Scabia. A partir de estos años se inicia un periodo oscuro en Volterra, que durará hasta la década de 1960, debido al marco político y social del país bajo el gobierno fascista. Esta situación se fue agravando con los sucesores de De Nigris, Umberto Sarteschi, a partir de 1939, y Gino Simonini, a principios de la década de 1950, quienes debieron lidiar con la crisis derivada de la Segunda Guerra Mundial (Fiorino, 2011, p.167). En el plano clínico, se respetaron las prácticas introducidas por De Nigris, a las que se fueron sumando las nuevas terapias farmacológicas.

En 1963 el Hospital de Volterra se unió al consorcio interprovincial formado por los institutos de Pisa y Livorno; y en 1978 inicia su clausura de acuerdo con la ley 180/1978 – Ley Basaglia –, pero no será definitiva hasta 1996 (Trovato, jun. 2021). Cuando se publica la ley 180/1978, la junta directiva del Hospital de Volterra contrató al fotógrafo local Piernello Manoni para documentar las pésimas condiciones en las que vivían los pacientes de la institución. Al observar el trabajo del fotógrafo (Trafeli, Trafeli, Manoni, 1985; I graffiti…, 2002), entendemos la progresiva degradación entre la época de Scabia, cuando el Hospital de Volterra pudo convertirse en paradigma de buenas prácticas psiquiátricas en Italia, y el periodo oscuro, cuando se torna hacia un aspecto manicomial, caracterizado por el encierro, el aislamiento y la decadencia de las instalaciones.

Manoni llevó a cabo su trabajo con diligencia, pero hizo algo más por el objeto de estudio que aquí abordamos: en el proceso de captar cada espacio del hospital con una mirada pausada y crítica, se topó con el mural de Nannetti, del cual quedó totalmente cautivado. Gracias a este encuentro fortuito hoy conocemos buena parte del mural, incluso con detalles, pues Manoni lo inmortalizó a través de su cámara pocos años después del traslado de Nannetti y antes de que comenzara a degradarse (Quinto, 2021, p.16-17).

## Palabras e imágenes en el espacio psiquiátrico

**Figura 1:**
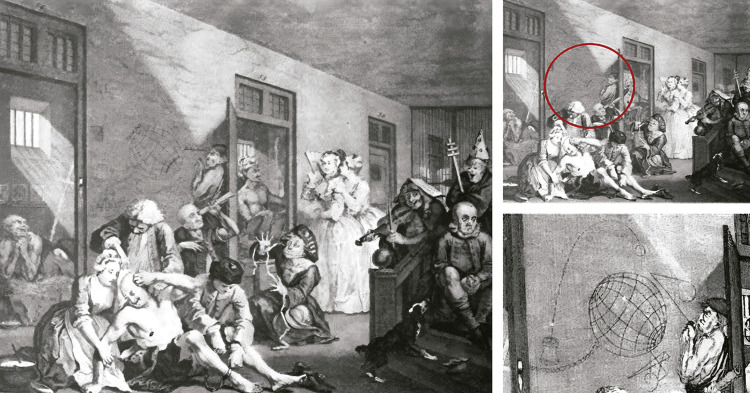
: William Hogarth, El manicomio, grabado, 1735 (MacGregor, 1989)

Las manifestaciones plásticas y la escritura han sido importantes recursos para aquellos y aquellas que dentro de los espacios psiquiátricos tenían la imperiosa necesidad de externalizar su mundo interior (Deeds, Goodman, Diamant, 2016). Entre los soportes utilizados para este fin, la pared es uno de los primigenios. Así lo vemos en grabados del siglo XVIII que representan asilos para locos donde un interno dibuja sobre la pared, como ocurre en la octava y última escena de la serie “El proceso del libertino” del artista británico William Hogarth (1697-1764) (Figura 1). A falta de documentación clínico-psiquiátrica hasta el siglo XIX, a finales de la década de 1980 el historiador del arte John MacGregor (1989, p.25-38) propuso utilizar estos grabados como fuente para rastrear los orígenes de un arte que se ha conocido por distintos nombres: arte psicopatológico, *art brut, outsider art, asylum art*, entre otras (Morgenthaler, 1992; Pereña, 2007; Bassan, 2009; Calicelli, 2010; García, 2010, 2018).

A partir de la segunda mitad del siglo XIX, este tipo de manifestaciones comenzaron a estudiarse desde un punto de vista científico; existe una importante literatura de la época que formuló grandes preguntas a su alrededor, de las cuales fue constante el interés por conocer los motivos que llevan a una persona afectada de una enfermedad o trastorno mental, y carente de cualquier formación educativa o artística, a acometer este tipo de manifestaciones – dibujo, pintura, escultura, escritura etc. – durante sus periodos de excitación, delirio o ingreso (Max Simon, 1876; Frigerio, 1880; Lombroso, Du Camp, 1880; Cristiani, 1897; Rogues de Fursac, 1905). Otra de las grandes cuestiones en torno a la que giraron estos estudios fue la elección de los materiales por parte del autor, si se trataba de azar o voluntad. En 1935, el neuropsiquiatra español Gonzalo Rodríguez Lafora propuso dos factores para este fenómeno: por un lado, la reclusión dentro del manicomio genera en el paciente una serie de contradicciones que precisan ser proyectadas hacia el exterior para mayor autocomprensión del autor; la segunda, se refiere a la enfermedad o trastorno como motor de un cambio en la personalidad (Trujillo Arrogante, 2023, p.4-5).

En este periodo, a la vertiente clínica se fue sumando de forma paulatina el acercamiento de algunos vanguardistas al arte realizado de los pacientes psiquiátricos, el cual valoraban por su potencial artístico. Entre estos, cabe citar el caso de los vanguardistas (Couet, 2017) y, ya en la década de 1940, el artista francés y creador del *art brut*, Jean Dubuffet (García, 2018).

En la década de 1970, contemporáneo al mural de Nannetti, José A. Escudero Valverde (1975, p.46) señaló que la implicación del paciente psiquiátrico en la creación pictórica se debe a un impulso que le obliga a prescindir del material o del lugar donde expresar sus creaciones.

Los estudios de las últimas décadas subrayan la relevancia de los dibujos y los escritos de los pacientes psiquiátricos no solo como documentos clínicos o como terapia, sino también como manifestaciones de la lucha por la identidad y la sanidad en contextos de reclusión psiquiátrica. Con ánimo de mencionar dos ejemplos, en 2016 se publicó *The electric pencil: drawings from inside State Hospital n.3*, un libro con los dibujos de James Edward Deeds realizados durante su internamiento en el Hospital de Missouri que se han convertido en un testimonio de la creatividad y resiliencia; y en el campo de la escritura, Ana Consiglieri y Olga Villasante (2024) han sido autoras del artículo “Las mujeres que escribieron en el Manicomio Nacional de Leganés tras la Guerra Civil Española, 1939-1952”, con el que exploran las cartas escritas por mujeres ingresadas en el Manicomio de Leganés (Madrid).

## El mural de Nannetti

Fernando Oreste Nannetti, carente de antecedentes artísticos, limitado por una escasa formación educativa, ingresado en distintas instituciones manicomiales a lo largo de su vida y sin participar en ningún programa de arteterapia, se lanzó a elaborar un gigantesco programa iconográfico que se extiende por buena parte del patio del pabellón Ferri. El mural mide 180 metros de longitud y dos de altura, y fue realizado durante nueve años, que se dividen en los dos periodos de ingreso en el pabellón Ferri: de 1959 a 1961 y de 1968 a 1973 (Trafeli, Peiry, Morandi, 2017). En el mural, Nannetti escribe y dibuja un contenido que, según afirma, recibe telepáticamente a través de torres – o antenas – de electricidad representadas en la misma pared. Es decir, nos da a entender que alguien, al otro lado de esas torres, transmite unos mensajes que él capta y reproduce en el gran lienzo pétreo (Quinto, 2021, p.11-13).

Desde el punto de vista estético, el mural se caracteriza por una marcada tosquedad, atribuida al único instrumento que el autor disponía para rayar la pared: el tirador de la cremallera de su chaqueta – en ocasiones lo sustituyó por pequeñas piedras. La elección de este instrumento obedece a, por un lado, prevenir heridas y lesiones al acometer las incisiones y, por otro, no tener que sustituir con frecuencia el “pincel” debido a su desgaste. La pared también puso de su parte, pues los materiales que la componen facilitaron el trabajo, una mezcla barata de arena y cal que permitía incidir sin mucho esfuerzo. Con todo, no restamos mérito técnico a nuestro autor, pues algunos fragmentos revelan un esfuerzo sostenido por repasar los perfiles grabados.

Entre el traslado de Nannetti y el cierre del Hospital, el recuerdo del mural permaneció en pocas personas, siempre del entorno hospitalario, especialmente en Aldo Trafeli y el fotógrafo Piernello Manoni, así como en los familiares de ambos. A ellos se debe la colaboración y promoción de varios proyectos dirigidos a difundir el mural más allá del pabellón Ferri, como son la película *L’osservatorio nucleare del Signor Nanof*, producida por Studio Azzurro, en 1985 (*L’osservatorio…*, 1985), el libro *N.O.F. 4: Il libro della vita*, en 1985 (Trafeli, Trafeli, Manoni, 1985), y el documental *I graffitti della mente*, en 2002 (I graffiti…, 2002).

Fruto de este trabajo de difusión, en 2011 el muralista italiano fue situado en la escena internacional del *art brut* a raíz de la exposición en la Colletion d’Art Brut de Lausana (Peiry, 2011; Sánchez Oms, 2011); y en 2013 nació la asociación Onlus Inclusione Grafio e Parola, fundada por los hijos y compañeros del celador, cuyo objetivo es elaborar estrategias para la restauración y conservación del mural. En su año de inauguración, la asociación organizó la recuperación de algunos fragmentos mediante su extracción de la pared, los cuales fueron depositados en el Centro de Documentación Lombroso de Volterra, ubicado en el área hospitalaria de San Lazzero y dependiente de la Autoridad Sanitaria Local de Pisa. En este centro, en 2015 se abrió un museo entre cuyas salas se colocaron algunos de los fragmentos del mural (Il museo…, 20 oct. 2015).

**Figura 2:**
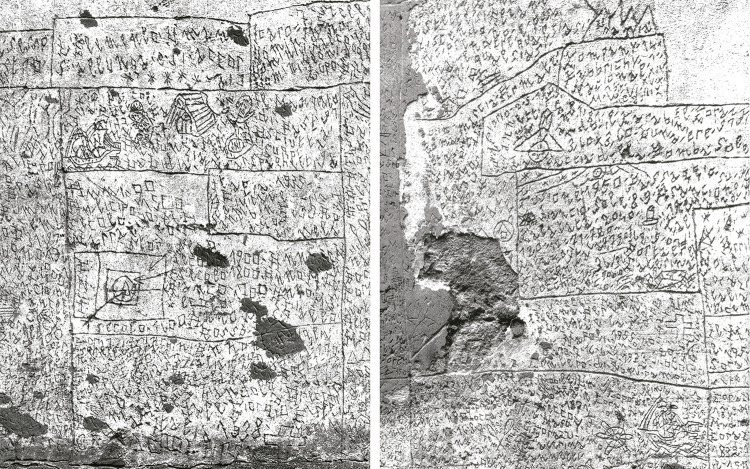
: Fragmentos del mural en el Pabellón Ferri de Volterra (Trafeli, Trafeli, Manoni, 1985)

El mural se compone de un conjunto de palabras e imágenes insertas en formas cuadradas o rectangulares y elaboradas con líneas rectas y sutiles curvas. Un tipo de producción que nos recuerda a los cartuchos egipcios. Estas formas generan una secuencia que, sin embargo, no es una narración encadenada y carece de hilo cohesivo; más bien se trata de mensajes que saltan de un fragmento a otro y se dispersan entre diversas secciones del mural (Figura 2).

**Figura 3:**
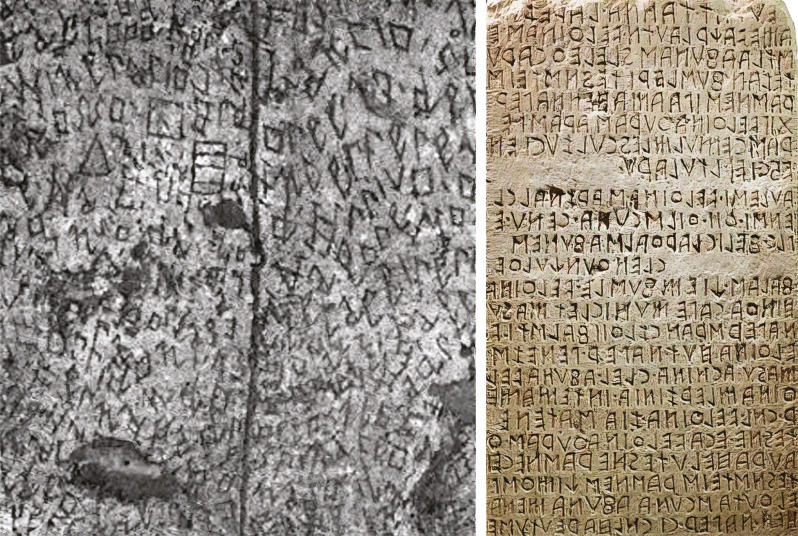
: Izquierda: fragmento del mural (Trafeli, Trafeli, Manoni, 1985); derecha: Cippo di Perugia (extraído del Museo Arqueológico Nacional de Umbría)

El texto de las formas geométricas se lleva a cabo mediante una tipografía ideada por el propio Nannetti: a rasgos generales, evita los trazos llenos y sueltos en favor de las mayúsculas angulosas, más fáciles de grabar. La estética resultante es evidente que tiene un asombroso parecido con las escrituras antiguas, en particular la demótica egipcia y la cuneiforme mesopotámica y, sobre todo, con la etrusca, lo que quizás esté relacionado con el ambiente volterrano y las actividades de arqueología organizadas por Fiumi (Figura 3).

El análisis del mural nos ofrece identificar sus rasgos principales: *horror vacui* – miedo al vacío o tendencia a rellenar todo el espacio – y un lenguaje ininteligible, caracterizado por párrafos cifrados, palabras fragmentadas y alteraciones de la estructura gramatical italiana que oscurecen el significado. Teniendo en cuenta los estudios que la profesora Ana Hernández Merino (2000, p.77-111) ha dedicado a este campo, podemos elaborar algunas interpretaciones sobre ambos aspectos en el caso de Nannetti: el *horror vacui* podría atribuirse a la preocupación del autor por maximizar el aprovechamiento del espacio en el que interviene; en cuanto a los signos irreconocibles en la obra, cabe pensar que Nannetti no se dirige a una comunidad o a un sujeto externo, sino a un espectador imaginario. Incluso, podría darse una situación de recelo hacia sus compañeros. Es decir, y como ya se ha comentado, Nannetti no se relacionaba con otros internos, y, según los testimonios extraídos del libro escrito por Trafeli, Peiry y Miorandi (2017), el contenido que el autor representa en el mural le llega de manera telepática, lo cual trataría de ocultar mediante un lenguaje encriptado por considerarlo una suerte de mensaje clasificado. Asimismo, introdujeron una perspectiva alternativa al plantear que el mural también sirve a Nannetti para afirmar su existencia ante el mundo, sin importarle si los demás son capaces de descifrar el mensaje (I graffiti…, 2002).

Aldo Trafeli actuó a modo de piedra Rosetta, ofreciendo valiosos datos sobre los temas representados en el mural.^2^ Antes de convertirse en celador del pabellón Ferri, Trafeli había estudiado Bellas Artes en Florencia. Llegó a Volterra con 27 años, para trabajar en el hospital psiquiátrico, y pronto se interesó por uno de los pacientes que no se relacionaba con nadie y sólo se dedicaba a dibujar en las paredes. Movido por la curiosidad y la sensibilidad artística, se acercó a Nannetti, con quien forjó la única amistad del muralista. Gracias a esta relación, el celador convenció al resto de sus compañeros para que permitieran a Nannetti continuar rayando las paredes del patio en el pabellón Ferri; de este modo, pasó mucho tiempo a su lado, aprendiendo a interpretar la obra (Quinto, 2021).

En diálogo con la palabra, la imagen; Nannetti elaboró un programa iconográfico dirigido a reforzar el texto, cuyo conjunto representa sus inquietudes en una amplia gama de signos y símbolos atravesados por un fuerte componente personal. Razón por la que Trafeli acertó en denominar la obra el “libro della vita” (Trafeli, Trafeli, Manoni, 1985). Los contenidos vienen representados hábilmente mediante una fusión de pictografía y anotaciones que, como aludimos antes, a veces se presentan en clave criptográfica. Un aparato concebido para hacer cuantificable y traducible la realidad del propio autor, a modo de una síntesis cohesiva que tiende puentes entre los marcos cognitivos internos y las representaciones externas.

Como se observa en las figuras anteriores, del mural brota una esencia primitivista caracterizada por símbolos muy pronunciados y una estructura compositiva simplificada. La presencia de elementos procedentes de civilizaciones antiguas en las manifestaciones artísticas de los pacientes psiquiátricos es otro de los grandes temas que interesaron en los primeros estudios sobre el campo. Así, se observa en no pocos autores de finales del siglo XIX y la primera mitad del XX, como los italianos Cesare Lombroso (1975) y Andrea Cristiani (1897) o el alemán Hans Prinzhorn (2012), entre otros. Con mayor o menor discrepancia, sobre estos autores voló el planteamiento de un proceso atávico, es decir, una suerte de regresión a fases primigenias del ser humano, de cuya fuente el paciente-autor extraería – o traería de vuelta – esa imaginería. Existen otras interpretaciones de la primera mitad del siglo XX, como los españoles José Pérez López-Villamil y Gonzalo Rodríguez Lafora, quienes se enfrentaron a casos clínicos – casi al mismo tiempo – en que los dibujos de un paciente presentaban rasgos análogos a las manifestaciones de algunas civilizaciones procedentes de la antigua Mesopotamia. Al principio, ambos barajaron distintas alternativas, como la idea del atavismo o la teoría junguiana sobre el inconsciente colectivo; en un principio, ninguno de los dos resolvió el enigma, pero pasados unos años retomaron los casos y acabaron descubriendo que sus pacientes habían estado expuestos a influencias de este género y posteriormente lo reflejaron en sus creaciones plásticas. En el caso que trató López-Villamil, el paciente había visto una exposición de arte persa hasta en dos ocasiones; en el caso de Lafora, el paciente estaba ingresado en Toledo y todos los años asistía a la procesión del *Corpus Christi*, momento en que las calles de alrededor de la catedral se cubren de tapices que poseen un marcado aire orientalista (Rodríguez Lafora, 1964; Angosto, 1985; Huertas, 2011).

En el trabajo de Nannetti cabría asociar la esencia primitivista a dos rasgos que ya hemos comentados: por un lado, los materiales y el soporte empleados otorgan a la obra una eminente tosquedad; por otro, el hospital está impregnado de la influencia etrusca del ambiente volteriano.

## Los contenidos del mural

El mural contiene una rica variedad de temas, como son la exposición de hechos futuros y hechos pasados – que solo suceden en la cabeza del autor –, creencias y religión, experiencias y sensaciones personales o conocimientos científicos inventados, entre otros (Trafeli, Trafeli, Manoni, 1996; Peiry, 2020). De todos, los dos últimos tienen un peso enorme en la biografía de Nannetti.

Los conocimientos sobre ciencia y tecnología son los más repetidos. La manera en que se representa podría compararse con el cuaderno de un científico o ingeniero de telecomunicaciones:

Telestaciones en conexión telepática directa. Misterio de las comunicaciones amplificadas mediante estetoscopio. Sneiz de televisión. Parato de televisión, aparato Macchi. La proyección de 16,34 es también ráfaga, 74 milímetros son cables dobles y transmisores de iones por medio de cuadrados y perfectos naturales. Antena atómica radio onda sonora con marco de acero inoxidable. Conexión de antenas de ondas gamma, radio y TV. Fuente de alimentación de haz magnético. Símbolos de Aníbal. Un cuerpo sólido vive en los espacios como un cuerpo en el agua y emite imágenes. El paso tachonado avanza por Europa. Avanza sin contrastes territoriales. 1961 Nannetti Fernando llega a Roma en Italia en helicóptero. Lanzamiento del misil a las 23h30 del 7 de octubre de 1960. Sobre México y Albania. La tierra se gangrena. Domingo 127. Lluvia de estrellas (I graffiti…, 2002).

Se trata de una ciencia ficticia, pero apoyada en una base razonable por dos motivos ya mencionados: la primera, su experiencia profesional como electricista en años precedentes a los ingresos institucionales; la segunda, las revistas de divulgación científica que Trafeli le proporcionaba. Todo ello explica el uso de terminología científica sin tener formación en el campo (Trafeli, Trafeli, Manoni, 1985). A lo largo de estos fragmentos, se despliegan ideas y descripciones que se dirigen a desarrollar, según el autor, un proyecto tecnológico desconocido. Además, el autor hace referencia a interlocutores con los que conversa y colabora para perfeccionar dicho proyecto.

Si atendemos a la esquizofrenia diagnosticada en Roma y a los estudios que Escudero Valverde (1975, p.65-66) dedicó a este campo, los esquizofrénicos suelen experimentar alteraciones del pensamiento caracterizadas por la incoherencia entre el “yo” y el “no yo” y su relación con la realidad. Esa disociación podría explorarse a través de las producciones artísticas, en las que el sujeto entrelaza elementos de la fantasía con facetas de la realidad.

En cuanto a la representación de experiencias vitales y sensaciones, sin olvidar que la mayor parte de su vida estuvo institucionalizada, aluden principalmente a la realidad del sistema clínico que le tocó vivir, para así contar, mediante un contraste de crudeza y delicadeza poética, los estados y las secuelas derivados de ciertos medicamentos y tratamientos que le eran aplicados. Así lo veremos en los fragmentos del mural analizados a continuación.

Llegados a este punto cabe citar algunos trabajos que han abordado la cuestión. Con ánimo de ser concisos, mencionamos a Shelley H. Carson (2011), cuyo modelo de vulnerabilidad compartida sugiere que la creatividad y los trastornos mentales comparten factores biológicos y cognitivos que facilitan tanto la creatividad mental como la psicopatología; por su parte, en las investigaciones sobre Carlos González Ragel, Mercedes Díaz Rodríguez (2013, 2006) argumenta que la creatividad no es necesariamente causada por la enfermedad mental, sino que son dos procesos autónomos que pueden coincidir en la misma persona. En ese sentido, la creatividad en un enfermo mental no surge de una elección consciente, sin embargo, en algunos casos, los artistas afectados por la esquizofrenia podrían encontrar una vía de expresión a través de las manifestaciones plásticas, plasmando sus visiones distorsionadas de la realidad. En un principio, Nannetti encajaría sobre todo en la descripción de Díaz Rodríguez; es decir, no se resta creatividad a su trabajo, sino que el motivo principal de estas manifestaciones es la exposición de sus vivencias e inquietudes, pero con un alto grado de distorsión de la realidad. Esto se observa bien en el tema sobre la ciencia y la tecnología, cuando el autor afirma que los contenidos le llegan mediante unas antenas receptoras. Sin embargo, ese conocimiento está relacionado con las revistas de divulgación científica y la profesión de electricista.

**Figura 4:**
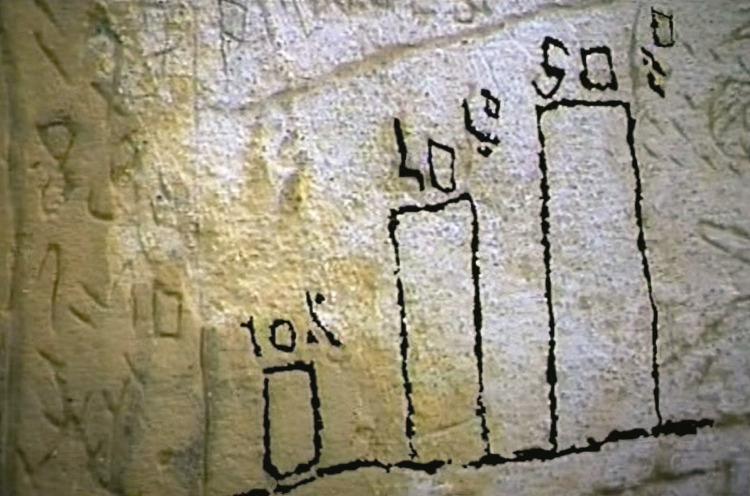
: Fragmento del mural. Captura de pantalla del documental I graffitti della mente (2002)

Aunque, en términos generales lo que tenemos delante es la prueba emocional de un paciente que lucha contra el miedo, la rabia, el dolor – físico y psicológico –, la indignación y el deseo. Incluso, llega a entender el origen de su enfermedad a partir de una serie de factores externos e internos, entre los que la psiquiatría sustenta el mayor porcentaje de las causas, como vemos en un gráfico representado en el mural (Figura 4): “10% por radiación magnética transmitida; 40% por enfermedad transmitida y provocada; 50% por odio personal, provocado o transmitido; causa de la muerte: choques catódicos y magnéticos, secreción medular, dislocaciones cardiovasculares, administración obligatoria de sustancias estupefacientes” (I graffiti…, 2002).^3^

Cuando describe las “causa de muerte”– choques catatónicos y magnéticos, secreción medular, dislocaciones cardiovasculares –, Nannetti probablemente se refiere a las repercusiones que en su cuerpo genera la terapia electroconvulsiva o electroshock – la primera aplicación de la historia se debe a Ugo Cerletti, en 1938, en Roma. En cuanto a la “administración obligatoria de sustancias estupefacientes” (Di Carlo, 2006; Trafeli, Trafeli, Manoni, 1985), Nannetti recurre varias veces al tema, pues en otros fragmentos del mural habla de sentirse narcotizado y sedado.

Este relato en primera persona constituye un testimonio que, en realidad, no concierne solo a Nannetti, sino a muchos pacientes psiquiátricos de la época, tanto fuera como dentro del Hospital de Volterra. Algo de lo que él mismo podría ser consciente. Incluso, quizás estos fragmentos simbolicen su forma de denunciar un sistema sanitario que no le proporciona remedios, sino más padecimientos. De hecho, las fechas en que se está ejecutando el mural coinciden con un momento clave en la historia de la medicina, cuando están creciendo las reacciones hacia la psiquiatría por movimientos contestatarios, agrupados en la conocida como “antipsiquiatría”, los cuales rechazaban las formas de violencia comunes en la institución manicomial tradicional, como el encierro forzoso, la represión, las prohibiciones o la aplicación de tratamientos nocivos, entre otros. Italia fue uno de los países más destacados en esta corriente, sobre todo por el entorno del psiquiatra Franco Basaglia (1924-1980), quien abogó por la transformación del sistema de salud mental en todo el país, proponiendo alternativas comunitarias y el tratamiento humanizado del paciente (Foot, 2014). Su gran hito se reflejó en la ya citada ley 180/1978, con la que “se inicia el proceso del cierre de los asilos, a la vez que se prevén herramientas para fortalecer actividades comunitarias, y toda una infraestructura de atención psiquiátrica con un servicio de Diagnóstico y Tratamiento en el Hospital General para atender urgencias y brindar apoyo y orientar la demanda a los centros de salud mental del territorio” (Huertas, Ortiz Lobo, 2021, p.56).

## Consideraciones finales

Los dibujos y los escritos de los pacientes psiquiátricos han sido ampliamente estudiados desde distintas disciplinas – arte, filosofía, psiquiatría, psicología etc. – y distintos puntos de vista. Las primeras aproximaciones al campo datan del siglo XIX y se centran en el análisis clínico; desde entonces, fue irrumpiendo el interés por otros aspectos de este, como son el creativo, el terapéutico o la capacidad autodescriptiva del autor o la autora, entendida esta última como un modo de mostrar las vivencias y las luchas tanto internas como externas.

En este marco de estudio, el mural de Fernando Oreste Nannetti (1927-1994) posee una importante conexión entre su biografía, el sistema sanitario italiano de la época y sus inquietudes o interés personales. A través de una técnica rudimentaria – rayar las paredes con un tirador de chaqueta – y una expresión que combina distintos lenguajes y una estética ciertamente personal – recuerda a escrituras de la antigüedad –, la obra muestra, por una parte, el testimonio de un paciente que narra, de manera codificada, sus vivencias ante las condiciones sanitarias y algunos tratamientos del sistema psiquiátrico que le tocó vivir; por la otra, la imaginación del autor, junto con sus experiencias y unos conocimientos básicos sobre ciencia, deriva en que la mayor parte de la superficie se dedique a un repertorio de referencias sobre proyectos tecnológicos inventados.

Estas características, tanto técnicas como estéticas y temáticas, otorgan un valor artístico al mural más allá del análisis clínico, lo que le ha valido de un reconocimiento internacional en línea del *art brut* o el *outsider art*. Asimismo, ha inspirado a otros artistas y directores de documentales, cuyas obras contribuyen a una mejor comprensión del mural.

## Data Availability

No están en repositorio.

## NOTAS

1 Movimiento artístico dirigido a ensalzar aquellas manifestaciones consideradas en los márgenes del discurso hegemónico o institucional del arte, sobre todo centrado en el arte realizado por los pacientes psiquiátricos, el cual fue el punto de partida para Jean Dubuffet.

2 Las transcripciones realizadas por Trafeli se encuentran en italiano dentro del libro *N.O.F. 4: il libro della vita* (Trafeli, Trafeli, Manoni, 1985) y el documental *I graffitti della mente* (2002).

3 Fragmento del mural traducido del italiano por el autor del presente artículo.
